# Supplementary material for: Devil in the details: how can we avoid potential pitfalls of CATS regression when our data do not follow a Poisson distribution?
Source: PeerJ. 2022 Jan 14;10:e12763. doi: 10.7717/peerj.12763 (PMC8763042; doi:10.7717/peerj.12763)
Supplement: Supplemental Information 2 [file peerj-10-12763-s002.pdf]

Appendix 2: Kullback-Leibler R^2 for members of exponential family

The following table summarizes the formulas of log-likelihood and Kullback-Leibler divergences, supposing that only location parameter (μ) is estimated, the scale parameter is known a priori (if it exists).

Distribution	Log-likelihood	Kullback-Leibler divergence
Gaussian (Normal)	$-n\ln(\sigma\sqrt{2\pi}) - \sum_{i=1}^{S}\frac{(y_i-\mu_i)^2}{2\sigma^2}$	$2\sum_{i=1}^{S}\frac{(y_i-\mu_i)^2}{2\sigma^2}$
Poisson	$\sum_i[y_i\ln(\mu_i)-\mu_i-\ln(y_i!)]$	$2\sum_{i=1}^{S}y_i\ln\left(\frac{y_i}{\mu_i}\right)$
Binomial	$\sum_i \ln\binom{n}{y_i}+y_i\ln\frac{\mu_i}{n}+(n-y_i)\ln\frac{n-\mu_i}{n}$	$2\sum_i y_i\ln\frac{y_i}{\mu_i}+(n-y_i)\ln\frac{n-y_i}{n-\mu_i}$
Negative binomial	$\sum_{i=1}^{S}\ln\frac{\Gamma(y_i+\theta)}{\Gamma(\theta)y_i!}-(y_i+\theta)\ln\left(\frac{\mu_i+\theta}{\theta}\right)+y_i\ln\frac{\mu_i}{\theta}$	$2\sum_{i=1}^{S}(y_i+\theta)\ln\left(\frac{\mu_i+\theta}{y_i+\theta}\right)+y_i\ln\frac{y_i}{\mu_i}$
Zero-truncated Poisson	$\sum_i[y_i\ln(\mu_i)-\mu_i-\ln(y_i!)-\ln(1-e^{-\mu_i})]$	$2\sum_i\left[y_i\ln\left(\frac{\mu_i^{full}}{\mu_i}\right)-\mu_i^{full}+\mu_i-\ln\left(\frac{1-\exp(-\mu_i)}{1-\exp(-\mu_i^{full})}\right)\right]$
Zero-truncated negative binomial	$\sum_{i=1}^{S}\ln\frac{\Gamma(y_i+\theta)}{\Gamma(\theta)y_i!}-(y_i+\theta)\ln\left(\frac{\mu_i+\theta}{\theta}\right)+y_i\ln\frac{\mu_i}{\theta}-\ln\left(1-\left(\frac{\mu_i+\theta}{\theta}\right)^{-\theta}\right)$	$2\sum_{i=1}^{S}(y_i+\theta)\ln\left(\frac{\mu_i+\theta}{\mu_i^{full}+\theta}\right)+y_i\ln\frac{\mu_i^{full}}{\mu_i}+\ln\left[\frac{1-\left(\frac{\mu_i+\theta}{\theta}\right)^{-\theta}}{1-\left(\frac{\mu_i^{full}+\theta}{\theta}\right)^{-\theta}}\right]$

Note that:

- In the case of binomial distribution: instead of usual parametrization (i.e., n = number of trials and p = probability of success) the parameters are n = number of trials, and $\mu = np$ = expected number of successes. n is assumed to be known a priori.
- Parameters of negative binomial distribution are mean (μ) and dispersion (θ). Variance is $V = \mu + \frac{\mu^2}{\theta}$. Dispersion is known a priori or value estimated by fitting the studied model is used.
- In deduction of KL-divergence of Poisson distribution we used the fact that for models containing intercept and using the canonical log-link $\sum\mu_i = \sum y_i$
- μ_i^{full} can be got by solving the $y_i = \frac{\mu_i^{full}}{1-\exp(-\mu_i^{full})}$ or $y_i = \frac{\mu_i^{full}}{1-\left(\frac{\mu_i^{full}+\theta}{\theta}\right)^{-\theta}}$ equations for zero-truncated Poisson and negative binomial distributions, respectively.

When canonical link is applied the predicted value of intercept-only model (μ^0) is equal to mean of y values ($\bar{y}$). Thus in this case

$$R_{KL}^2 = 1 - \frac{\sum_{i=1}^{n}\left(y_i - \mu_i\right)^2}{\sum_{i=1}^{n}\left(y_i - \bar{y}\right)^2}$$

for Gaussian (normal) distribution,

$$R_{KL}^2 = 1 - \frac{\sum_{i=1}^{s} y_i \ln\left(\frac{y_i}{\mu_i}\right)}{\sum_{i=1}^{s} y_i \ln\left(\frac{y_i}{\bar{y}}\right)}$$

for Poisson distribution,

$$R_{KL}^2 = 1 - \frac{\sum_i y_i \ln\frac{y_i}{\mu_i} + \left(n - y_i\right)\ln\frac{n - y_i}{n - \mu_i}}{\sum_i y_i \ln\frac{y_i}{\bar{y}} + \left(n - y_i\right)\ln\frac{n - y_i}{n - \bar{y}}}$$

for binomial distribution, and

$$R_{KL}^2 = 1 - \frac{\sum_{i=1}^{S}\left(y_i + \theta\right)\ln\left(\frac{\mu_i + \theta}{y_i + \theta}\right) + y_i \ln\frac{y_i}{\mu_i}}{\sum_{i=1}^{S}\left(y_i + \theta\right)\ln\left(\frac{\bar{y} + \theta}{y_i + \theta}\right) + y_i \ln\frac{y_i}{\bar{y}}}$$

for negative binomial distribution.

In the two-stage models, the expected value of y_i depends on two parameters: the probability of presence (*p*) and expected value if y_i is positive (μ). In contrast to dispersion or power parameters, we cannot assume that the value of *p* is known a priori; it is always estimated from the data and often depends on the independent variables. Therefore, it has to be included in the general form of the log-likelihood function:

$$l(\mathbf{p}, \boldsymbol{\mu}; \mathbf{y}) = \sum_{i=1}^{s}(1 - y_i^+)\ln(1 - p_i) + y_i^+ \ln p_i + \sum_{y_i > 0} \ln f^+(y_i; \mu_i) \qquad (1)$$

where $f^+(y_i; \mu_i)$ is the density function of the distribution fitted to positive data, and y_i^+ is the presence/absence of species

$$y_i^+ = \begin{cases} 1 & \text{if } y_i > 0 \\ 0 & \text{otherwise} \end{cases}$$

Since the likelihood depends on both $\mathbf{p}$ and $\boldsymbol{\mu}$, the definition of R^2 given in equation (20) of the main text should be replaced by:

$$R_{KL}^2 = 1 - \frac{l(\mathbf{p}^{\mathbf{full}}, \boldsymbol{\mu}^{\mathbf{full}}; \mathbf{y}) - l(\mathbf{p}, \boldsymbol{\mu}; \mathbf{y})}{l(\mathbf{p}^{\mathbf{full}}, \boldsymbol{\mu}^{\mathbf{full}}; \mathbf{y}) - l(\boldsymbol{p}^{\mathbf{0}}, \boldsymbol{\mu}^{\mathbf{0}}; \mathbf{y})} \tag{2}$$

where $l(\mathbf{p}^{\mathbf{full}}, \boldsymbol{\mu}^{\mathbf{full}}; \mathbf{y})$ and $l(\boldsymbol{p}^{\mathbf{0}}, \boldsymbol{\mu}^{\mathbf{0}}; \mathbf{y})$ are log-likelihoods of the full and intercept-only models, respectively. Intercept-only models mean that only intercepts are used as predictors in both stages.

Two-stage models can be fitted as two separate GLMs. Let us denote the likelihood of the two fitted models by $l(\mathbf{p}; \mathbf{y})$ and $l(\boldsymbol{\mu}; \mathbf{y})$. It can be shown that:

$$l(\mathbf{p}, \boldsymbol{\mu}; \mathbf{y}) = l(\mathbf{p}; \mathbf{y}) + l(\boldsymbol{\mu}; \mathbf{y}) \tag{3}$$

In a full model, the predicted and observed values are the same, so $l(\mathbf{p}^{\mathbf{full}}; \mathbf{y}) = 0$ and $l(\mathbf{p}^{\mathbf{full}}, \boldsymbol{\mu}^{\mathbf{full}}; \mathbf{y}) = l(\boldsymbol{\mu}^{\mathbf{full}}; \mathbf{y})$.

Positive abundances can be modeled by beta, zero-truncated Poisson, and zero-truncated negative binomial distributions. In the case of a beta distribution, $\ln f^+(y_i; \mu_i)$ is the log-likelihood of a beta distribution fitted for positive abundances (see Table 1 for formula). In beta regression, the formula given in Table 1 can be used for calculating $l(\boldsymbol{\mu}; \mathbf{y})$. Since the predicted value of the intercept-only model may differ from the mean of observed values, it has to be fitted before calculation of $l(\boldsymbol{\mu}^{\mathbf{0}}; \mathbf{y})$.

In the case of zero-truncated Poisson and zero-truncated negative binomial distributions,

$$f^+(y_i; \mu_i) = \frac{f(y_i; \mu_i)}{1 - f(0; \mu_i)} \tag{4}$$

where $f(y_i; \mu_i)$ is the density of Poisson or negative binomial distributions. In truncated distributions, the expected value is a nonlinear function of μ:

$$E(y|y > 0, \mu) = \frac{\mu}{1 - f(0; \mu)} \tag{5}$$

where $f(0;\mu_i) = e^{-\mu}$ for a zero-truncated Poisson distribution and $f(0;\mu_i) = (1+\mu/\theta)^{-\theta}$ for a zero-truncated negative binomial distribution. Therefore, for the calculation of $l(\boldsymbol{p}^{\mathbf{0}}, \boldsymbol{\mu}^{\mathbf{0}}; \mathbf{y})$, the intercept-only model should be fitted. If the dispersion parameter (ϕ or θ in beta or negative binomial model, respectively) is estimated during regression, the estimated value should be used in calculation of the log-likelihood for full and intercept-only models.
