# Supplementary material for: Devil in the details: how can we avoid potential pitfalls of CATS regression when our data do not follow a Poisson distribution?
Source: PeerJ. 2022 Jan 14;10:e12763. doi: 10.7717/peerj.12763 (PMC8763042; doi:10.7717/peerj.12763)
Supplement: Supplemental Information 3 [file peerj-10-12763-s003.pdf]

Appendix 2 Equivalence with formula from Shipley (2014)

For a Poisson model without offset, the Kullback-Leibler R^2 is (Table 1):

$$R_{KL}^2 = 1 - \frac{\sum_{i=1}^{s} y_i \ln\left(\frac{y_i}{\mu_i}\right)}{\sum_{i=1}^{s} y_i \ln\left(\frac{y_i}{\bar{y}}\right)} \tag{1}$$

It can be re-written in the form of equation (4) from Shipley (2014) using the observed and expected relative abundance ($o_i = y_i/y_{tot}$ and $p_i = \mu_i/y_{tot}$, respectively) and number of species:

$$R_{KL}^2 = 1 - \frac{\sum_{i=1}^{s} \frac{y_i}{y_{tot}} \ln\left(\frac{y_i/y_{tot}}{\mu_i/y_{tot}}\right)}{\sum_{i=1}^{s} \frac{y_i}{y_{tot}} \ln\left(\frac{y_i/y_{tot}}{\bar{y}/y_{tot}}\right)} = 1 - \frac{\sum_{i=1}^{s} o_i \ln\left(\frac{o_i}{p_i}\right)}{\sum_{i=1}^{s} o_i \ln\left(\frac{o_i}{1/S}\right)} \tag{2}$$
