# Supplementary material for: Devil in the details: how can we avoid potential pitfalls of CATS regression when our data do not follow a Poisson distribution?
Source: PeerJ. 2022 Jan 14;10:e12763. doi: 10.7717/peerj.12763 (PMC8763042; doi:10.7717/peerj.12763)
Supplement: Supplemental Information 4 [file peerj-10-12763-s004.pdf]

Appendix 3: $\boldsymbol{R^2_{adj}}$ is a special case of $\boldsymbol{R^2_{KL,adj}}$ for Gaussian distribution

For a Gaussian distribution, the scaled deviances of the fitted and intercept-only models are:

$$D^*(\mathbf{y}, \boldsymbol{\mu}) = \frac{\sum(y_i - \mu_i)^2}{\hat{\sigma}^2} = \frac{SS_E}{\hat{\sigma}^2}$$

$$D^*\left(\mathbf{y}, \boldsymbol{\mu}^{\mathbf{0}}\right) = \frac{\sum(y_i - \bar{y})^2}{\hat{\sigma}^2} = \frac{SS_T}{\hat{\sigma}^2} \tag{1}$$

Since σ^2 is unknown, its unbiased estimate is used in the formula:

$$\hat{\sigma}^2 = \frac{SS_E}{n-k-1} \tag{2}$$

The adjusted Kullback-Leibler R^2 is:

$$\boldsymbol{R^2_{KL,adj}} = \frac{\frac{SS_T}{\hat{\sigma}^2} - \frac{SS_E}{\hat{\sigma}^2} - k}{\frac{SS_T}{\hat{\sigma}^2}} = \frac{SS_T - SS_E - \hat{\sigma}^2 k}{SS_T} \tag{3}$$

Substituting (2) into (3), we obtain the commonly used formula for adjusted R-squared:

$$R^2_{KL,adj} = \frac{SS_T - SS_E - \frac{kSS_E}{n-k-1}}{SS_T} = 1 - \frac{SS_E}{SS_T}\left(1 + \frac{k}{n-k-1}\right) = 1 - (1 - R^2)\frac{n-1}{n-k-1} \tag{4}$$
